# Supplementary material for: Alterations in the Human Plasma Lipidome in Response to Tularemia Vaccination
Source: Vaccines (Basel). 2020 Jul 24;8(3):414. doi: 10.3390/vaccines8030414 (PMC7564507; doi:10.3390/vaccines8030414)
Supplement: Supplementary file 1 [file vaccines-08-00414-s001.zip › vaccines-756931-suppl.pdf]

vaccines

MDPI

Article

Alterations in the Human Plasma Lipidome in Response to Tularemia Vaccination

Supplementary Table S1. *Subject Demographics.*

Category	Sub-Category	DVC-LVS (N=10)
Gender	Female	5 (50%)
	Male	5 (50%)
Race	Black / African American	3 (30%)
	White	7 (70%)
Ethnicity	Non-Hispanic or Non-Latino	10 (100%)
Age	Mean (S.D.)	27.3 (6.3)
	Median	26
	Range	22.7, 44.1

Supplementary Table S2. ***Table of Lipidomics Standards.*** The following synthetic standards were used to optimize instrumental parameters and also for quantification of patient plasma samples.

**OXY Standards**		
Lipid Abbrev.	Lipid Species	Conc. (pg/ml)
OEA	Oleoylethanolamide	14.2
AEA	Arachidonoylethanolamine	28.4
PGE2 EA	Prostaglandin E2 Ethanolamide	11.4
PGF2a EA	Prostaglandin F2a Ethanolamide	14.2
	12(13)-EpOME/13-HODE	
12(13) EpOME	12(13)epoxy-9-octadecenoic acid	1.1
13 HODE	13-hydroxy-9,11-octadecadienoic acid	1.4
9,10 DIHOME	9,10-dihydroxy-12-octadecenoic acid	1.1
TBX2	Thromboxane B2	11.4
12 HETE	12-hydroxy-5,8,10,14-eicosatetraenoic acid	1.1
	9-HETE/11(12)-EET	
9 HETE	9-hydroxy-5,7,11,14-eicosatetraenoic acid	1.1
11(12) EET	11(12)-epoxy-5,8,14-eicosatrienoic acid	1.1
20 HETE	20-hydroxy-5,8,11,14-eicosatetraenoic acid	1.1
5 HETE	5-hydroxy-6,8,11,14-eicosatetraenoic acid	1.1
8(9) EET	8(9)-epoxy-5,11,14-eicosatrienoic acid	1.1
	14(15)-epoxy-5,8,11-eicosatrienoic acid	1.1
	Summed DHET Species	
14,15 DHET	14,15-dihydroxy-5,8,11-eicosatrienoic acid	1.1
11,12 DHET	11,12-dihydroxy-5,8,14-eicosatrienoic acid	1.1
8,9 DHET	8,9-dihydroxy-5,11,14-eicosatrienoic acid	1.1
5,6 DHET	5,6-dihydroxy-8,11,14-eicosatrienoic acid	1.1

**Shotgun Lipidomics Standards**		
Lipid	Lipid Class	[Conc; ug/ul]

di20:0 PC	Phosphatidylcholine	60.0
di14:0 PE	Phosphatidylethanolamine	1.0
17:0 SM	Spingomyelin	4.0
di20:0 DG	Diacylglyerol	4.0
17:0 FFA	Free Fatty Acid	1.0
Tri17:1 TG	Triacylglycerol	10.0
di14:0 PS	Phosphatidylserine	0.1
17:0 LPC	Lysophosphatidylcholine	2.5
14:0 LPE	Lysophosphatidylethanolamine	0.1

Supplementary Table S3. ***Table of instrumental parameters.*** A list of optimized instrumental parameters used to conduct targeted lipidomics studies at EMO and SLU laboratories, respectively.

**EMO Instrumental Parameters**			
Curtain Gas (CUR)	25.00	Collision Gas (CAD)	Low
ESI Voltage (IS)	-3500	Gas Source 1 (GS1)	55.00
ESI Temp (TEM)	650°C	Gas Source 2 (GS2)	50.00
Declustering Potential (DP)	-90.00	Collision Energy (CE)	-40.00
Entrance Potential (EP)	-10.00	Collision Energy Spread (CES)	0.00
Q1 and Q3 Resolution	Unit	Step Size	0.2Da

**SLU Instrumental Parameters**			
Curtain Gas (CUR)	20.00	Sheath Gas	12 arb. units
ESI Voltage (IS)	3500	Ion Sweep Gas	1 arb. Units
Capillary Temp.	270°C	Auxillary Gas	2 arb. units
Collision Pressure (Argon)	1 atm	Capillary Offset	35

Supplementary Table S4. *Table of serum cytokines serum samples.* A list of the cytokines measured and represented in Figure 6.

**Figure 6 Cytokines and Abbreviations**					
EOTAXIN	basic fibroblast growth factor (FGF-BASIC)	granulocyte colony-stimulating factor (G CSF)	granulocyte-macrophage colony stimulating factor (GM CSF)	interferon gamma (IFN-γ)	tumor necrosis factor alpha (TNF-a)
interleukin 10 (IL-10)	interleukin 10 (IL-10)	interleukin 13 (IL-13)	interleukin 17 (IL-17)	interleukin 1 receptor agonist (IL-1RA)	vascular endothelial growth factor (VEGF)
interleukin 2 (IL-2)	interleukin 4 (IL-4)	interleukin 5 (IL-5)	interleukin 8 (IL-8)	interleukin 9 (IL-9)	interleukin 10 (IP-10)

monocyte chemoattractant protein-1/monocyte chemotactic and activating factor (MCP-1/MCAF)	macrophage inflammatory protein 1b (MIP 1B)	platelet derived growth factor BB (PDGF BB)	RANTES

Supplementary Table S5. ***Table of positively identified targeted lipids in serum samples.*** A list of all of the identified targeted lipids are shown in this table with corresponding mass to charge (m/z) value and lipid scan type/experiment.

Tularemia Lipid ID	Lipid Species	Scan	m/z
TULIPID001	OEA	MRM	326.0
TULIPID002	AEA	MRM	348.0
TULIPID003	PGE2 Ethanolamide	MRM	378.0
TULIPID004	PGF2a Ethanolamide	MRM	380.0
TULIPID005	12(13)-EpOME; 13-HODE	MRM	169.0
TULIPID006	9,10 DIHOME	MRM	173.0
TULIPID007	Summed DHET Species	MRM	NA
TULIPID008	TXB2	MRM	147.0
TULIPID009	12-HETE	MRM	87.0
TULIPID010	9-HETE; 11(12)-EET	MRM	167.0
TULIPID011	20-HETE	MRM	289.0
TULIPID012	5-HETE	MRM	301.0
TULIPID013	8(9)-EET	MRM	70.0
TULIPID014	14(15)-EET	MRM	220.0
TULIPID015	14:0 LPC	NL50	502.5
TULIPID016	15:0 LPC	NL50	516.4
TULIPID017	16:1 LPC	NL50	528.4
TULIPID018	16:0 LPC	NL50	530.4
TULIPID019	17:0 LPC	NL50	544.4
TULIPID020	18:2 LPC	NL50	554.4
TULIPID021	18:1 LPC	NL50	556.4
TULIPID022	18:0 LPC	NL50	558.4
TULIPID023	20:4 LPC	NL50	578.4
TULIPID024	20:3 LPC	NL50	580.4
TULIPID025	22:6 LPC	NL50	602.4
TULIPID026	22:5 LPC	NL50	604.3
TULIPID027	30:2 PC	NL50	736.7
TULIPID028	30:1 PC	NL50	738.7
TULIPID029	30:0 PC	NL50	740.6
TULIPID030	32:1e PC	NL50	752.7
TULIPID031	32:0e PC	NL50	754.9
TULIPID032	32:1 PC	NL50	766.7
TULIPID033	32:0 PC	NL50	768.6
TULIPID034	34:1p PC	NL50	778.7
TULIPID035	34:0p PC	NL50	780.6
TULIPID036	34:3 PC	NL50	790.7
TULIPID037	34:2 PC	NL50	792.7
TULIPID038	34:1 PC	NL50	794.7
TULIPID039	34:0 PC	NL50	796.7
TULIPID040	36:3p PC	NL50	802.7
TULIPID041	36:4 PC	NL50	816.6
TULIPID042	36:3 PC	NL50	818.7
TULIPID043	36:2 PC	NL50	820.7
TULIPID044	36:1 PC	NL50	822.7

TULIPID045	38:4p PC	NL50	828.6
TULIPID046	38:6 PC	NL50	840.6
TULIPID047	38:5 PC	NL50	842.6
TULIPID048	38:4 PC	NL50	844.7
TULIPID049	38:3 PC	NL50	846.7
TULIPID050	38:2 PC	NL50	848.8
TULIPID051	38:1 PC	NL50	850.7
TULIPID052	38:0 PC	NL50	852.6
TULIPID053	40:0e PC	NL50	866.8
TULIPID054	40:6 PC	NL50	868.7
TULIPID055	40:5 PC	NL50	870.7
TULIPID056	40:0 PC	NL50	880.8
TULIPID057	34:2 PI	PREC241	833.4
TULIPID058	34:1 PI	PREC241	835.5
TULIPID059	36:4 PI	PREC241	857.7
TULIPID060	36:2 PI	PREC241	861.6
TULIPID061	36:1 PI	PREC241	862.8
TULIPID062	38:5 PI	PREC241	883.6
TULIPID063	38:4 PI	PREC241	885.7
TULIPID064	38:3 PI	PREC241	887.0
TULIPID065	38:2 PI	PREC241	888.0
TULIPID066	16:0 Cer	NL256	536.6
TULIPID067	18:1 Cer	NL256	562.0
TULIPID068	18:0 Cer	NL256	564.6
TULIPID069	20:1 Cer	NL256	590.0
TULIPID070	20:0 Cer	NL256	592.6
TULIPID071	22:1 Cer	NL256	618.6
TULIPID072	22:0 Cer	NL256	620.7
TULIPID073	23:0 Cer	NL256	634.7
TULIPID074	24:2 Cer	NL256	644.8
TULIPID075	24:1 Cer	NL256	646.6
TULIPID076	24:0 Cer	NL256	648.7
TULIPID077	14:0 CE	NL368	619.7
TULIPID078	16:1 CE	NL368	645.7
TULIPID079	16:0 CE	NL368	647.7
TULIPID080	18:3 CE	NL368	669.7
TULIPID081	18:2 CE	NL368	671.7
TULIPID082	18:1 CE	NL368	673.7
TULIPID083	18:0 CE	NL368	675.8
TULIPID084	20:5 CE	NL368	693.7
TULIPID085	20:4 CE	NL368	695.7
TULIPID086	22:6 CE	NL368	719.7
TULIPID087	22:5 CE	NL368	721.7
TULIPID088	22:4 CE	NL368	723.7
TULIPID089	22:3 CE	NL368	725.0
TULIPID090	22:2 CE	NL368	727.8
TULIPID091	e32:0 PE	FMOC	898.7
TULIPID092	32:2 PE	FMOC	908.6
TULIPID093	32:1 PE	FMOC	910.7
TULIPID094	32:0 PE	FMOC	912.8
TULIPID095	p34:2 PE	FMOC	920.8
TULIPID096	p34:1 PE	FMOC	922.7
TULIPID097	e34:1 PE	FMOC	924.8
TULIPID098	34:3 PE	FMOC	934.9
TULIPID099	34:2 PE	FMOC	936.8
TULIPID100	34:1 PE	FMOC	938.8
TULIPID101	p36:4 PE	FMOC	944.8
TULIPID102	e36:4 PE	FMOC	946.8

TULIPID103	e36:3 PE	FMOC	948.8
TULIPID104	e36:2 PE	FMOC	950.8
TULIPID105	36:5 PE	FMOC	958.7
TULIPID106	36:4 PE	FMOC	960.7
TULIPID107	36:3 PE	FMOC	962.8
TULIPID108	36:2 PE	FMOC	964.8
TULIPID109	36:1 PE	FMOC	966.8
TULIPID110	36:0 PE	FMOC	968.8
TULIPID111	p38:5 PE	FMOC	970.8
TULIPID112	p38:4 PE	FMOC	972.8
TULIPID113	e38:4 PE	FMOC	974.8
TULIPID114	38:6 PE	FMOC	984.7
TULIPID115	38:5 PE	FMOC	986.8
TULIPID116	38:4 PE	FMOC	988.8
TULIPID117	38:3 PE	FMOC	990.8
TULIPID118	p40:7 PE	FMOC	994.8
TULIPID119	p40:6 PE	FMOC	996.8
TULIPID120	p40:5 PE	FMOC	998.8
TULIPID121	40:7 PE	FMOC	1010.9
TULIPID122	40:6 PE	FMOC	1012.8
TULIPID123	40:5 PE	FMOC	1014.7
TULIPID124	40:4 PE	FMOC	1016.8
TULIPID125	40:1 PE	FMOC	1022.8
TULIPID126	16:1 SM	NL50	735.6
TULIPID127	16:0 SM	NL50	737.6
TULIPID128	18:1 SM	NL50	763.6
TULIPID129	18:0 SM	NL50	765.6

© 2020 by the authors. Licensee MDPI, Basel, Switzerland. This article is an open access article distributed under the terms and conditions of the Creative Commons Attribution (CC BY) license (http://creativecommons.org/licenses/by/4.0/).
